# Comparative evaluation of the aggregate index of systemic inflammation (AISI) and its modified version for early detection of central line-associated bloodstream infection: a pilot study using machine learning techniques

**DOI:** 10.3205/dgkh000661

**Published:** 2026-06-30

**Authors:** Gargee Anand, Ketan Priyadarshi, Bandana Kumari, Jutang Babat Ain Tiewsoh, Rijhul Lahariya

**Affiliations:** 1 Department of Microbiology, All India Institute of Medical Sciences, Patna, Bihar, India; 2 Department of Microbiology, All India Institute of Medical Sciences, Deoghar, Jharkhand, India; 3 Department of Biochemistry, All India Institute of Medical Sciences, Patna, Bihar, India; 4 All India Institute of Medical Sciences, Patna, Bihar, India

**Keywords:** central line-associated bloodstream infection, CLABSI, aggregate index of systemic inflammation, AISI, early prediction, machine learning

## Abstract

**Background:**

Central line-associated bloodstream infections (CLABSI) are a common and serious problem in critically ill patients; their early detecting is challenging. This study evaluated the predictive ability of the aggregate index of systemic inflammation (AISI) and its modified form for early identification of CLABSI within two calendar days following central line insertion, using a machine learning approach.

**Method:**

We conducted an analysis of patients who received central line insertion. Inflammatory indices were calculated using laboratory parameters obtained on second day post-insertion. Four machine learning algorithms were applied to evaluate their predictive performance for early CLABSI detection.

**Results:**

Among 234 patients who met the inclusion criteria, 39 were confirmed CLABSI cases. We found both indices significantly elevated in the CLABSI group. Modified AISI demonstrated the strongest performance using XGBoost, with the highest area under the ROC curve (0.99), 97% sensitivity and 98% specificity, indicating its potential as the better early screening marker for CLABSI than AISI.

**Conclusion:**

Both AISI and modified AISI demonstrated strong predictive value for early CLABSI detection, being both accessible and cost-effective. Modified AISI outperformed AISI in predictive performance. These findings support the need for the prospective validation of the modified AISI before clinical implementation.

## Zusammenfassung

**Hintergrund:**

Zentralvenenkatheter (ZVK)-assoziierte Blutstrominfektionen (CLABSI) stellen bei kritisch kranken Patienten ein häufiges und schwerwiegendes Problem dar; ihre frühzeitige Erkennung ist eine Herausforderung. In dieser Studie wurde die Vorhersagekraft des Aggregate Index of Systemic Inflammation (AISI) und seiner modifizierten Form für die frühzeitige Erkennung von CLABSI innerhalb von zwei Kalendertagen nach Katheterlegung unter Verwendung eines maschinellen Lernansatzes untersucht.

**Methode:**

Wir führten eine Analyse bei Patienten durch, bei denen ein ZVK gelegt wurde. Die Entzündungsindizes wurden anhand von Laborwerten berechnet, die am zweiten Tag nach Katheteranlage erhoben wurden. Vier Algorithmen des maschinellen Lernens wurden eingesetzt, um die Vorhersageleistung zur frühzeitigen Erkennung von CLABSI zu bewerten.

**Ergebnisse:**

Von 234 Patienten, die die Einschlusskriterien erfüllten, waren 39 bestätigte CLABSI-Fälle. Wir stellten fest, dass beide Indizes in der CLABSI-Gruppe signifikant erhöht waren. Der modifizierte AISI zeigte unter Verwendung von XGBoost die beste Leistungsfähigkeit mit der höchsten Fläche unter der ROC-Kurve (0,99), einer Sensitivität von 97% und einer Spezifität von 98%, was auf sein Potenzial als besserer Marker für das Früherkennen von CLABSI im Vergleich zum AISI hinweist.

**Schlussfolgerung:**

Sowohl der AISI als auch der modifizierte AISI zeigten einen hohen prädiktiven Wert für die Früherkennung von CLABSI, erwiesen sich als leicht zugänglich und kosteneffizient. Das modifizierte AISI übertraf AISI hinsichtlich der prädiktiven Leistungsfähigkeit. Die Ergebnisse untermauern die Notwendigkeit einer prospektiven Validierung vor der klinischen Einführung des modifizierte AISI.

## Introduction

Healthcare-associated infections (HAIs), particularly central line-associated bloodstream infections (CLABSIs) are particularly concerning due to their rapid progression and high impact on patient outcomes [1], [2]. CLABSIs are associated with extended intensive care unit (ICU) stays, increased morbidity and mortality and significant financial costs to health systems globally [3], [4]. These infections can escalate swiftly to severe complications such as sepsis or multi-organ dysfunction, emphasizing the critical need for timely diagnosis [5]. Blood cultures are currently the cornerstone for diagnosing bloodstream infections [6]. However, their clinical utility is constrained by delayed processing times and limited sensitivity in early infection phases. Such limitations may lead to overtreatment and contribute to antimicrobial resistance [6], [7]. Therefore, identifying rapid, accurate and cost-effective biomarkers for early CLABSI detection remains a vital unmet clinical need.

Systemic inflammatory markers from routine blood tests, particularly the complete blood count (CBC) and the systemic inflammatory response index (SIRI), are increasingly studied as early indicators of infection [8], [9]. Ratios like the neutrophil-to-lymphocyte ratio (NLR) and platelet-to-lymphocyte ratio (PLR) have shown promise in various infections but are not well established for early CLABSI detection [10], [11], [12]. After central venous catheter (CVC) insertion, local injury to the vessel wall triggers a systemic immune response [13]. Neutrophils increase due to delayed cell death driven by inflammatory signals, while lymphocyte levels drop as part of stress-related immune suppression [14], [15]. Monocytes temporarily leave the bloodstream to enter tissues and aid in the immune response [16]. Platelets are activated by bacterial toxins and release cytokines that further drive inflammation [17]. In addition, albumin levels fall during inflammation due to reduced production and increased leakage from blood vessels [18]. As a negative acute-phase protein, albumin reflects both the severity of inflammation and the patient’s nutritional status [19].

These immune-hematological shifts underpin the rationale for composite indices such as the aggregate index of systemic inflammation (AISI), which incorporates neutrophil, monocyte and platelet counts relative to lymphocytes to reflect the host's inflammatory state [20]. While AISI has demonstrated prognostic utility in sepsis, cardiovascular disease and malignancy, its role in early detection of CLABSIs remains uninvestigated. Recognizing that hypoalbuminemia is a common and clinically significant marker of systemic inflammation and poor prognosis in critically ill patients, we propose a modified version of AISI, calculated by dividing the traditional AISI by serum albumin, to enhance its sensitivity and specificity. 

Given that both CBC and albumin levels are routinely available within hours of ICU admission, this study aims to evaluate and compare the predictive performance of AISI and modified AISI for early CLABSI detection within two calendar days of central line insertion.

## Materials and methods

### Patient selection and data collection

This observational study was conducted among adult patients admitted to the ICU. From a total of 850 patients aged over 18 years who had a CVC placed in 2024, a statistically adequate sample size of 260 was calculated using the Taro Yamane formula, assuming a 5% margin of error and a 95% confidence level. The study population included all ICU patients aged 18 years or older who had a central line in place for more than two consecutive calendar days. Patients who presented with documented bloodstream infections at the time of admission were excluded. Case identification and infection surveillance were conducted in accordance with the standardized criteria of the Centers for Disease Control and Prevention’s National Healthcare Safety Network (NHSN) [21]. All patient information was anonymized before analysis by removing any identifying details.

Basic demographic variables (age and sex) and a vital clinical measure (body temperature) were recorded on the second day after central line insertion, along with blood-based inflammatory indices. These variables were chosen for their routine clinical availability, relevance to systemic inflammatory processes and low variability in measurement. Collecting only essential and easily available data makes this study practical and suitable for use in resource-limited settings, where access to advanced tests may be limited. We excluded other procedural confounders (catheter site and duration) due to low event rates, adhering to the events-per-variable (EPV) rule to prevent overfitting. Our analysis prioritized early CBC-derived inflammatory indices to reduce bias and ensure applicability in resource-limited settings. The calculation of inflammatory indices based on CBC included the following (20):

AISI: neutrophils (%) × platelets (thousands/microlitre) × monocytes (%) / lymphocytes (%)

Modified AISI: AISI / albumin (gram/decilitre)

### Statistical analysis

Data was analyzed using SPSS version 26. The Shapiro-Wilk test was used to check if continuous variables followed a normal distribution. Variables were expressed as mean ± standard deviation (SD) when normally distributed and as median with interquartile range (IQR) when not. Categorical variables were summarized as counts and percentages. Patients were divided into two groups, CLABSI-positive and CLABSI-negative, and comparisons between them were made using the Independent t-test or Mann-Whitney U test for continuous variables, based on normality and the Chi-square test for categorical data. Relationships between inflammatory indices and continuous clinical variables were examined using either Pearson’s or Spearman’s correlation, depending on data distribution. Multicollinearity among predictors was checked using the Variance Inflation Factor (VIF). A p-value of ≤0.05 was considered statistically significant and marked with an asterisk (*).

**Figure 1:**
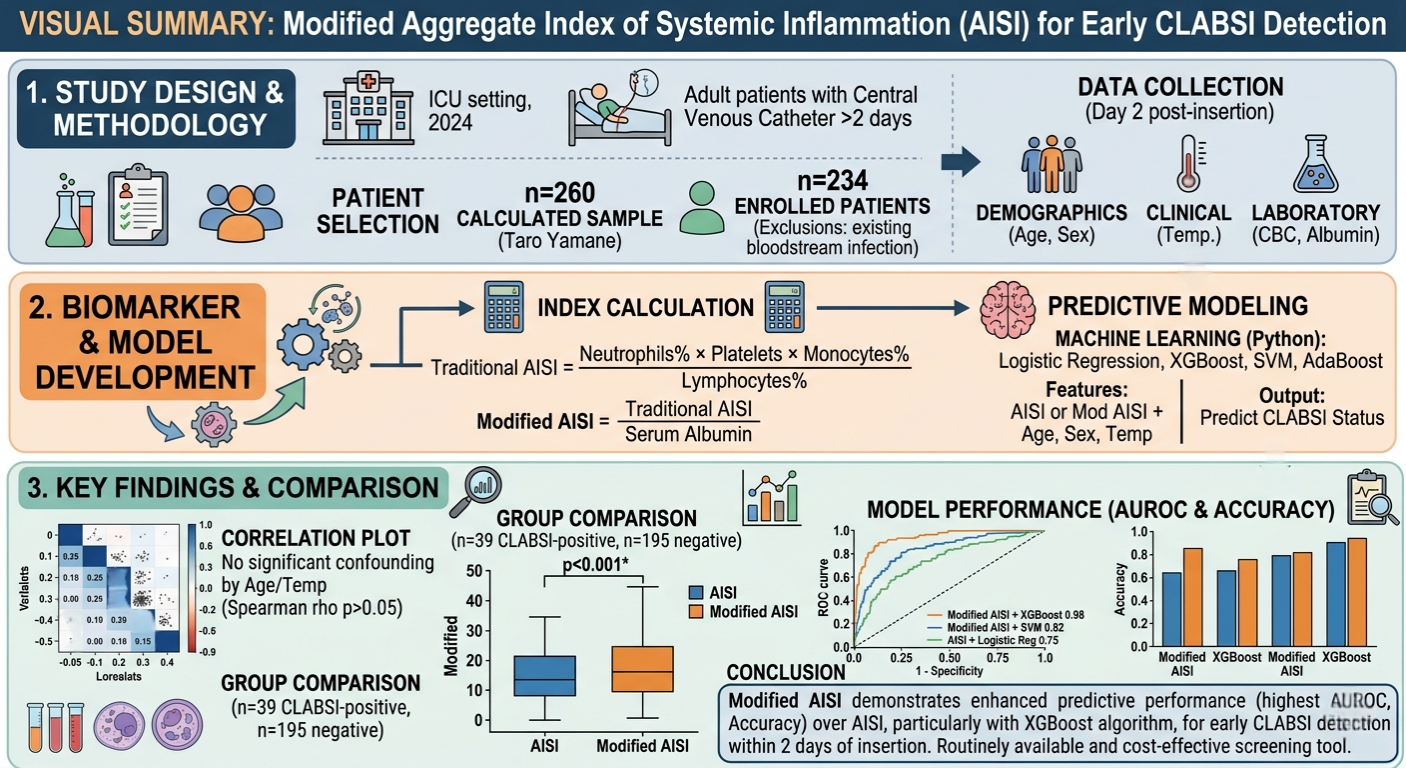
Summarising the methodology and the clinical workflow of the findings of this study.

To evaluate the predictive capability of CBC-derived indices—AISI and modified AISI—machine learning (ML) models were developed to predict the occurrence of CLABSI. Four primary models were developed: Logistic Regression, eXtreme Gradient Boosting (XGBoost), Support Vector Machines (SVM) and Adaptive Boosting (AdaBoost), using these indices along with key clinical variables (age, sex, and temperature) as input features. The dataset was split into a 70:30 training-to-test ratio to ensure proper evaluation. Each model's performance was assessed using standard classification metrics, including accuracy, sensitivity, specificity, positive predictive value (PPV) and negative predictive value (NPV) with their 95% confidence interval. The area under the receiver operating characteristic curve (AUROC) was calculated to evaluate the discriminative ability of each model. All the ML algorithms were written in the Python scripting language (version 3.10.12, Python Software Foundation, Wilmington, DE, USA). Various libraries were used to create plots, ML algorithms, for data handling and numerical operations (Figure 1 (Fig. 1)).

## Results

A total of 234 patients were enrolled in the study, meeting the inclusion criteria. The median age of the cohort was 55 years (IQR: 41.3–67.0), with a higher proportion of male patients (58.5%). A comparative analysis of clinical and laboratory variables between CLABSI-positive and CLABSI-negative patients was performed and the baseline characteristics of the cohort are summarized in Table 1 (Tab. 1). In the CLABSI-positive group, the median age was 55 years (IQR, 39.5–65), while the median age in the CLABSI-negative group was 56 years (IQR, 42.5–68).

**Figure 2:**
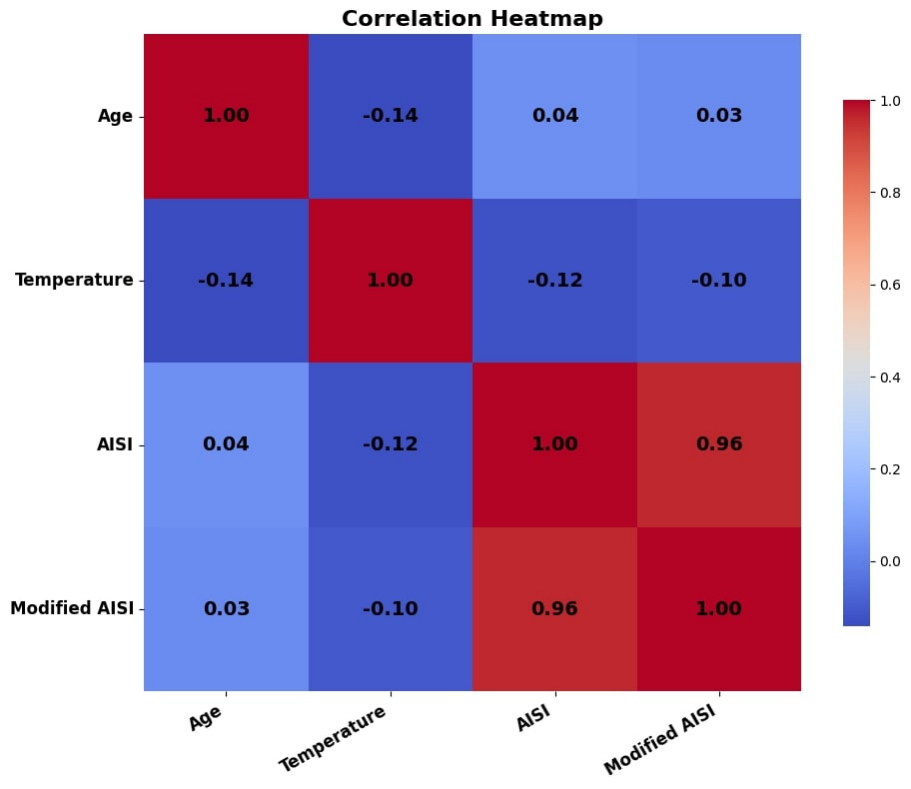
Correlation coefficient of the variables assessing the confounder.

While age, sex and temperature did not show significant differences between the CLABSI-positive and negative groups, the inflammatory indices, including AISI and the modified AISI, demonstrated notable variations. These indices combine multiple immune cell values, allowing them to capture subtle changes that might not be apparent when assessing individual cell types in isolation. This integrated approach provides a more sensitive and comprehensive view of the body's early immune response to infection, especially with the introduction of the modified AISI. Prior to building model, we first examined the potential confounding effects of age and temperature on the inflammatory indices. This was done by assessing their correlations with each index using the Spearman rank correlation test. The results showed no significant correlations, suggesting that age and temperature were not strongly associated with the inflammatory indices (Figure 2 (Fig. 2)).

We further checked for the predictive power of AISI and modified AISI for CLABSI using four different ML algorithms, with the CLABSI-negative group set as the reference. Additionally, we assessed performance using several metrics, including accuracy, sensitivity, specificity, PPV, NPV and AUROC, which revealed distinct differences in the performance of the models for AISI and modified AISI (Table 2 (Tab. 2)). To mitigate potential biases arising from the relatively small sample size, bootstrapping was performed to resample the dataset and enhance the stability and generalizability of the model performance metrics. For AISI, logistic regression demonstrated a relatively lower performance, with high sensitivity but lower specificity and accuracy. In contrast, models like XGBoost and SVM showed much higher performance, especially in terms of accuracy, with high sensitivity and specificity, making them more reliable in distinguishing between positive and negative outcomes. 

**Figure 3:**
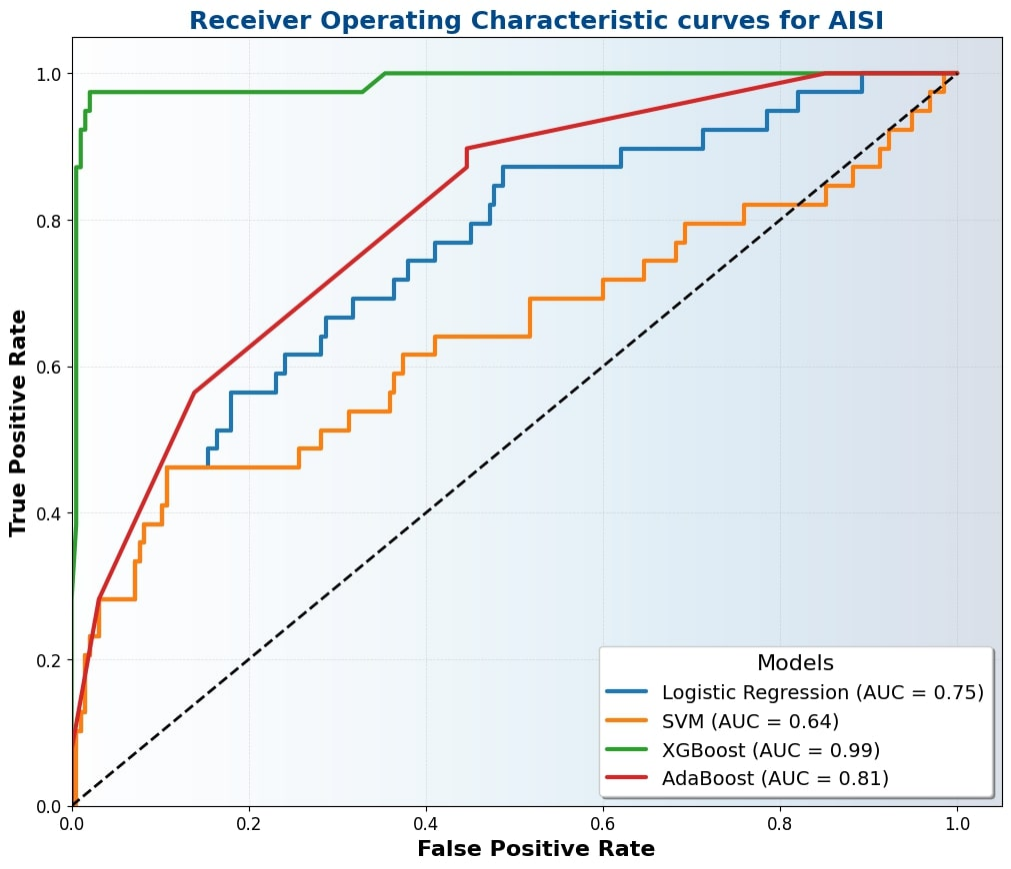
Receiver Operating Characteristic curves comparing the diagnostic performance of AISI among various ML algorithms.

**Figure 4:**
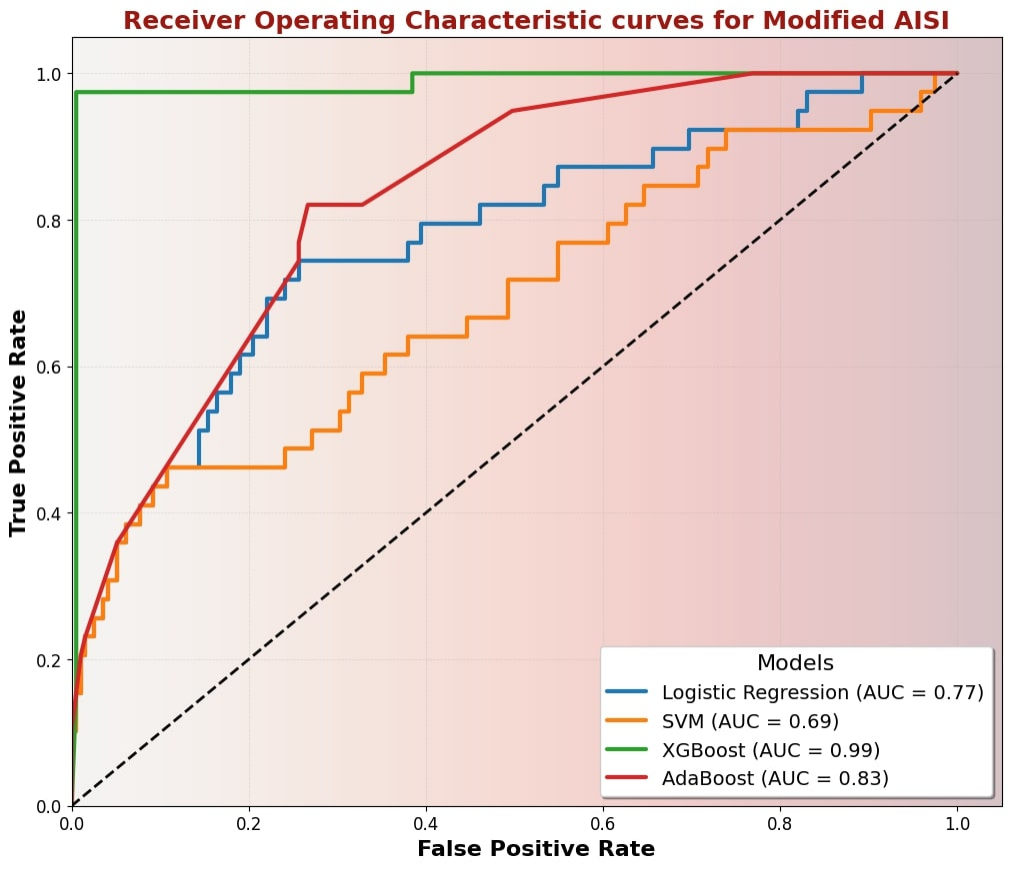
Receiver Operating Characteristic curves comparing the diagnostic performance of modified AISI among various ML algorithms.

For modified AISI, both XGBoost and SVM maintained excellent performance, with accuracy near 100%, indicating their robustness in predicting the outcomes. Logistic regression and AdaBoost showed moderate improvements in modified AISI but were still less efficient compared to the other models. In summary, while modified AISI generally enhanced model performance, XGBoost and SVM consistently outperformed other models in both scenarios. In all cases, modified AISI's enhanced predictive power made it a more reliable and effective marker, underscoring its potential as a more robust biomarker for the early detection of CLABSI compared to AISI. Figure 3 (Fig. 3) and Figure 4 (Fig. 4) shows the comparative ROC curves of various ML algorithms for AISI and modified AISI, respectively.

## Discussion

CLABSI remain a significant cause of morbidity and prolonged hospitalization in critically ill patients, highlighting the need for reliable early detection methods [1]. CLABSI demand the most urgent attention than other HAIs, as it carries a burden comparable to the eighth leading cause of death in the United States [22]. Traditional diagnostic approaches, which often rely on clinical suspicion and basic blood parameters, can fail to identify subtle immune changes during the early stages of infection [6]. This study examined the use of composite inflammatory indices, AISI and modified AISI, which integrate multiple immune cell counts to improve detection. While age, sex and temperature showed no significant differences between CLABSI-positive and negative groups, both AISI and modified AISI exhibited substantial differences, with modified AISI demonstrating a stronger performance. Machine learning models, particularly XGBoost (0.99) and SVM (0.82), showed superior predictive accuracy with modified AISI as compared to Logistic Regression (0.74) and AdaBoost (0.75), indicating its enhanced potential for early CLABSI detection.

The insertion of a CVC causes a disruption in the endothelial lining, triggering a localized inflammatory response that increases the risk of infection [13]. This breach allows for the attachment of pathogens to the catheter surface and the formation of biofilms [23]. In response, the innate immune system activates rapidly: neutrophils are recruited as the first line of defense, releasing reactive oxygen species (ROS), enzymes, and forming neutrophil extracellular traps (NETs) to contain the spread of infection [24]. Monocytes are also attracted to the site, differentiating into macrophages that secrete pro-inflammatory cytokines [25]. Platelets are activated simultaneously, adhering to the damaged blood vessel and pathogens, releasing mediators that enhance immune cell recruitment and promote clot formation [26]. Endothelial cells upregulate adhesion molecules to facilitate leukocyte trafficking, while lymphocyte activity may be modulated, often showing transient depletion or redistribution as part of the systemic stress response [27].

AISI integrate various immune cell counts to provide a more comprehensive view of the immune status [28]. The modified AISI index further strengthens this approach by incorporating albumin levels, a key biomarker that reflects the body's nutritional status and inflammatory response. Albumin, being an acute-phase protein, decreases in response to inflammation, making it a useful addition to inflammatory indices [18]. The integration of albumin with immune cell counts in the modified AISI allows for a more robust and nuanced measure of immune response, potentially improving the early detection of infections. Machine learning models applied to these indices, such as XGBoost and SVM, can further enhance predictive accuracy. 

In several studies, AISI has shown strong potential as a prognostic indicator across various inflammatory and infectious conditions, including odontogenic abscesses, COVID-19, sepsis and hypertension [20], [28], [29], [30], [31]. Studies demonstrate its superior predictive accuracy for disease severity and mortality compared to traditional markers like CRP or individual leukocyte ratios [29],[30]. Although very few studies have specifically evaluated AISI in CLABSI, its consistent performance in related systemic infections suggests promise for early detection and risk stratification in CLABSI [9], [32]. Incorporating albumin into the modified AISI may further enhance its sensitivity to inflammation and machine learning models can refine its predictive utility in clinical settings. As the NHSN defines CLABSI as a surveillance term, these biomarkers should be used only for case classification and not to guide antimicrobial therapy. Treatment decisions should be based on both clinical and microbiological assessments. We only looked at results from the first two calendar days after insertion, so using this test beyond this period would require further study with repeated measurements.

To the best of our knowledge, this is the first study to explore both AISI and modified AISI in the context of CLABSI. By incorporating ML algorithms for better evaluation, this study provided new insights about these accessible and hematology-based biomarkers for early detection, within 2 calendar days of central line insertion. These readily available and cost-effective markers could facilitate timely interventions, but there are certain limitations. First, the retrospective nature of data collection introduces selection bias, which may affect the predictive performance. Second, the small sample size of a single center, primarily due to our eligibility criteria and our centre’s relatively low prevalence of CLABSI. Despite this, the final sample has enough power for preliminary statistical analyses, may slightly impact the generalizability of the findings. Future studies should also assess whether using AISI and modified AISI to detect CLABSI early, can improve patient outcomes, such as reducing ICU stay or avoiding unnecessary antibiotics, to understand their true value in clinical practice.

## Conclusion

This study highlights that inflammatory index, especially AISI and its modified version, can be useful for early detection of CLABSI within two calendar days after central line insertion. Both indices showed good predictive ability using the XGBoost algorithm, but the modified AISI had the best performance, with the highest AUROC, accuracy, sensitivity and specificity. These indices are based on routine blood tests and easy to calculate, making them practical tools for early screening and triaging. Further large-scale prospective studies are needed to confirm these findings and to explore their usefulness in different clinical settings and patient populations.

## Notes

### Authors’ ORCIDs

- Anand G: https://orcid.org/0009-0008-0473-389X
- Priyadarshi K: https://orcid.org/0000-0003-4623-3523
- Kumari B: https://orcid.org/0000-0001-5395-413X
- Tiewsoh JBA: https://orcid.org/0000-0003-0782-6588
- Lahariya R: https://orcid.org/0009-0003-5769-4509

### Ethical approval 

The study was approved by the Institutional Ethics Committee of All India Institute of Medical Sciences, Patna.

### Funding

None. 

### Acknowledgements

We acknowledge the contributions of all authors to the conception, drafting, and critical revision of this original article.

### Authors contribution

Ketan Priyadarshi, Gargee Anand and Rijhul Lahariya contributed to the conception and design of this manuscript. Rijhul Lahariya, Gargee Anand and Ketan Priyadarshi were responsible for the methodology and initial draft preparation. Literature search and data collection were conducted by Rijhul Lahariya, Bandana Kumari, Jutang Babat Ain Tiewsoh and Gargee Anand. The final draft was written by Rijhul Lahariya, Gargee Anand and Ketan Priyadarshi. All authors reviewed, revised, and approved the final manuscript. 

Anand G and Priyadarshi K contributed equally to this work.

### Ethical approval

This study was approved by the Institutional Ethics Committee of All India Institute of Medical Sciences, Patna (Ref.No. AIIMS/Pat/IEC/UG-STS/MBBS 2021/ Dec24/15). The requirement for informed consent was waived by the committee. All data were anonymized to maintain patient confidentiality, and the study was conducted in accordance with the ethical standards of the Declaration of Helsinki.

### Competing interests

The authors declare that they have no competing interests.

## Figures and Tables

**Table 1:**
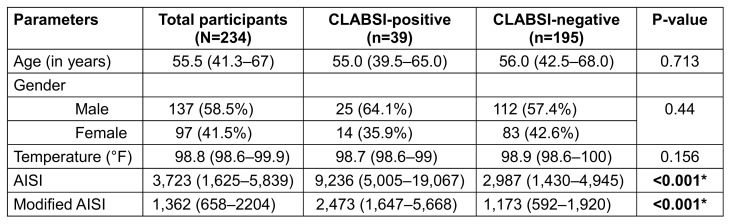
Demographic, clinical and inflammatory indices among CLABSI-positive and negative patients (N=234).

**Table 2:**
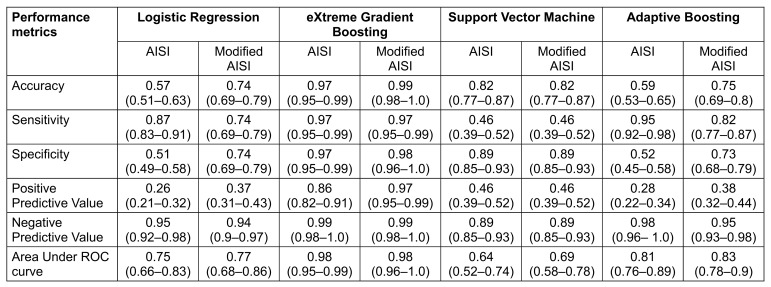
Performance metrics of AISI and modified AISI in predicting CLABSI using various machine learning algorithms.
